# Crosslinked Fibroin Nanoparticles: Investigations on Biostability, Cytotoxicity, and Cellular Internalization

**DOI:** 10.3390/ph13050086

**Published:** 2020-04-30

**Authors:** Duy Toan Pham, Nuttawut Saelim, Raphaël Cornu, Arnaud Béduneau, Waree Tiyaboonchai

**Affiliations:** 1 Faculty of Pharmaceutical Sciences, Naresuan University, Phitsanulok 65000, Thailand; phamd58@email.nu.ac.th (D.T.P.); nut456zz@hotmail.com (N.S.); 2 PEPITE EA4267, Univ. Bourgogne Franche-Comté, F-25000 Besançon, France; raphael.cornu@univ-fcomte.fr (R.C.); arnaud.beduneau@univ-fcomte.fr (A.B.); 3 The Center of Excellence for Innovation in Chemistry (PERCH-CIC), Department of Chemistry and Center of Excellence for Innovation in Chemistry, Faculty of Science, Mahidol University, Bangkok 10400, Thailand; 4 The Center of Excellence for Innovation in Chemistry (PERCH-CIC), Naresuan University, Phitsanulok 65000, Thailand

**Keywords:** fibroin, nanoparticles, crosslink, cellular uptake, biostability

## Abstract

Recently, crosslinked fibroin nanoparticles (FNP) using the crosslinker 1-ethyl-3-(3-dimethylaminopropyl) carbodiimide hydrochloride (EDC) or the polymer poly(ethylenimine) (PEI) have been developed and showed potentials as novel drug delivery systems. Thus, this study further investigated the biological properties of these crosslinked FNP by labeling them with fluorescein isothiocyanate (FITC) for in vitro studies. All formulations possessed a mean particle size of approximately 300 nm and a tunable zeta potential (−20 to + 30 mV) dependent on the amount/type of crosslinkers. The FITC-bound FNP showed no significant difference in physical properties compared to the blank FNP. They possessed a binding efficacy of 3.3% *w*/*w*, and no FITC was released in sink condition up to 8 h. All formulations were colloidal stable in the sheep whole blood. The degradation rate of these FNP in blood could be controlled depending on their crosslink degree. Moreover, no potential toxicity in erythrocytes, Caco-2, HepG2, and 9L cells was noted for all formulations at particle concentrations of < 1 mg/mL. Finally, all FNP were internalized into the Caco-2 cells after 3 h incubation. The uptake rate of the positively charged particles was significantly higher than the negatively charged ones. In summary, the crosslinked FNP were safe and showed high potentials as versatile systems for biomedical applications.

## 1. Introduction

Fibroin, a fibrous protein commonly extracted from the domestic silkworm *Bombyx mori* silk, has gained increasing attention and focused research in various biomedical applications [1,2,3,4,5,6,7]. Amongst several platforms such as films [8,9], hydrogels [10,11,12], tablets [13], scaffolds [14,15,16], and microparticles [17,18], fibroin nanoparticles (FNP) are the most utilized systems for drug delivery purposes [4,19,20]. In most studies, unmodified FNP are popularly formulated using desolvation method, in which the aqueous solution of regenerated fibroin is mixed homogeneously with an organic solvent to yield insoluble submicron particles [19,21,22,23]. This method creates unmodified FNP with obvious limitations that may hinder their versatility in clinical uses. Firstly, unmodified self-assembly FNP have loose and uncompact structure, with hydrogen bonding and hydrophobic interactions as their main stabilizing forces [24]. As a consequence, this reduces the entrapment efficiency and loading capacity of the encapsulated drugs that bind with FNP via non-covalent bonds [25,26]. Additionally, due to the weak interactions, these particles commonly release the drugs in a rapid and uncontrolled manner [21,27,28]. Secondly, as fibroin is a negatively charged protein at pH 7.0, the surface charge of unmodified FNP is therefore negative [29]. The fact that the cell membrane also possesses a negative charge hinders the versatility of the system, especially in cellular binding, internalization, and mucoadhesiveness properties [30].

To overcome the mentioned FNP disadvantages, we developed crosslinked FNP using the crosslinkers 1-ethyl-3-(3-dimethylaminopropyl) carbodiimide hydrochloride (EDC) or poly(ethylenimine) (PEI) [24,25,26]. These novel systems successfully enhanced the entrapment efficiency and loading capacity of both model drugs α-mangostin [25] and paclitaxel [26], whereas the mean particle size remained similar to the non-crosslinked FNP. Interestingly, the FNP surface zeta potential could be adjusted favorably, from negative (−20 mV) to positive (+ 30 mV), by varying the amount of EDC or PEI [24]. Lastly yet importantly, the crosslinked FNP controlled the encapsulated drug release rate in a sustained manner, dependent on the amount of crosslinker [25,26].

These crosslinked FNP, although proving potential in enhancing FNP properties for drug delivery, have not been systematically investigated for their biostability, cytotoxicity, and in vitro cellular interactions. It is commonly acknowledged that the positively charged entities are highly toxic to the cells, especially the red blood cells [31,32]. Moreover, the positively charged PEI polymer, a popular gene transfecting agent, is lethal in some cell lines, dependent on its molecular weight and structure [31,33,34]. In addition, the cellular internalization, generally preferred as the ability to be taken up by the cells by endocytosis, either by receptor-mediated endocytosis, caveolae-dependent endocytosis, macropinocytosis, or phagocytosis, is crucial for the nanoparticle studies. To this end, the particle surface charges might play an important role in cellular internalization of FNP [32]. Furthermore, although most FNP applications are used intravenously, the biostability of these particles in the systemic circulation (i.e., blood) has been limited explored. The ability to control the FNP degradability rate is crucial as it determines both the drug pharmacokinetics and pharmacodynamics. Thus, by crosslinking fibroin, we hypothesized that the crosslinked FNP could be more enzymatically stable than the non-crosslinked ones due to their increase in fibroin β-sheet content.

Taking aforementioned issues into account, in this study, we formulated crosslinked FNP utilizing EDC and PEI and compared with non-crosslinked FNP on various aspects. Fluorescein isothiocyanate (FITC) was used as a fluorescence dye to track the particles in cellular studies. All formulations were then characterized in terms of mean size, polydispersity index (PI), zeta potentials, FITC-binding efficacy and release profiles, and physical stability. The biostability in whole blood of all formulations was conducted using mass loss measurement. Additionally, the cytotoxicity of the FNP was performed in the red blood cells and various cell lines, namely 9L, Caco-2, and HepG2. Finally, the cellular internalization studies on Caco-2 were conducted both qualitatively and quantitatively.

## 2. Results and Discussion

### 2.1. FNP Characterization

To track the particles in biological systems, FNP were labeled with FITC, a xanthene dye. FITC is the most popularly used fluorescence labelling agent for cell-based experiments due to its water solubility, ease of conjugation, high quantum efficiency, and low nonspecific binding with biological systems [35]. Generally, FITC isothiocyanate groups could react with fibroin residual amine groups to form thioamide (thiourea) covalent bonds. However, the success of this process depends on various stringent factors such as fibroin concentrations and buffers. Moreover, the incomplete removal of unreacted FITC might cause high background fluorescence in cellular studies. Thus, thorough characterizations should be considered.

**Figure 1:**
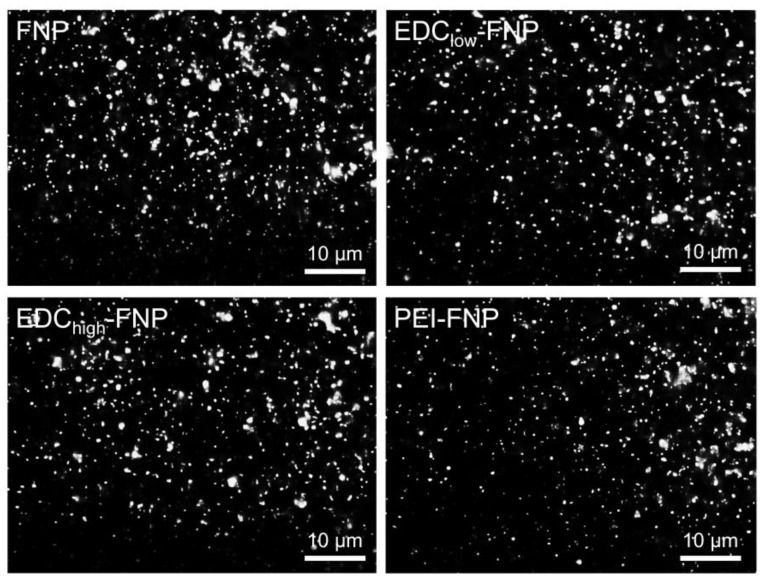
Fluorescence images of fluorescein isothiocyanate (FITC)-bound fibroin nanoparticle formulations. Scale bar: 10 µm.

Our results demonstrated that FITC-bound FNP were successfully prepared with homogeneous distribution in the medium (Figure 1). In addition, no free dye staining the image background was observed, suggesting that the unbound FITC was completely washed off. All formulations manifested similar FITC-binding efficiency of approximately 3.5% *w*/*w*, with no statistically significant difference. These qualitative results were in agreement with FITC dissolution studies. After 8 h in the sink condition at 37 °C, less than 0.5% of FITC was released from all FNP formulations.

In terms of physical properties, Table 1 summaries the mean size, PI, and zeta potential of four investigated FNP formulations, including non-crosslinked FNP, EDC_low_-FNP, EDC_high_-FNP, and PEI-FNP. No significant difference between the blank FNP and the FITC-bound FNP was noted, indicating that the FITC incorporation did not affect the particle properties. All formulations possessed similar particle size of approximately 300 nm and an acceptable size distribution with PI of < 0.3. On the other hand, the surface zeta potential of the non-crosslinked FNP and EDC_low_-FNP had negative values of −18 mV, which is the normal charge of fibroin in water, whereas the EDC_high_-FNP and PEI-FNP had positive values of + 30 mV. Thus, the positively charged polymer PEI and the high amount of EDC successfully altered the FNP surface charge from negative to positive [24]. Moreover, all formulations were stable at 4 °C for at least 6-month storage, with no significant differences in mean particle size and zeta potential.

### 2.2. Biostability and Hemolysis Study

Intravenous injection is one of the most common applications of nanoparticles [23]. Thus, the interaction between FNP and the circulatory blood components critically affects their efficiency. Fibroin has been proved by the U.S. Food and Drug Administration (FDA) as a biomaterial, and its film formulations are hemocompatible [36]. However, limited studies have explored this issue on FNP. To this end, we reported three set of data, including the biostability in the whole blood, the degradation behaviors, and the hemolysis ability to the erythrocytes of four investigated FNP.

Generally, due to their high kinetics energy, most nanoparticles tend to aggregate in the biological systems before contacting the cells. These aggregates might result in blockage of the blood vessel and in altering the nanoparticle properties [37]. Thus, we first investigate the FNP colloidal stability in the cell culture media. The result showed that all FNP were aggregated within 1 h in the Dulbecco’s Modified Eagle Medium (DMEM) medium without fetal bovine serum (FBS). Interestingly, in the complete medium with FBS, although their zeta potential was modified to become near 0 mV, they were colloidal stable for 3 days with no significant changes in particle size (data not shown). Similar result was observed when incubating FNP in the whole blood at 37 °C: all formulations were stable for 24 h without observable aggregation. Therefore, the superior colloidal stability of FNP in whole blood could be a result from stabilizing effects of serum protein, such as albumin, coating on the particle surface, which help prevent particle aggregation [38].

**Figure 2:**
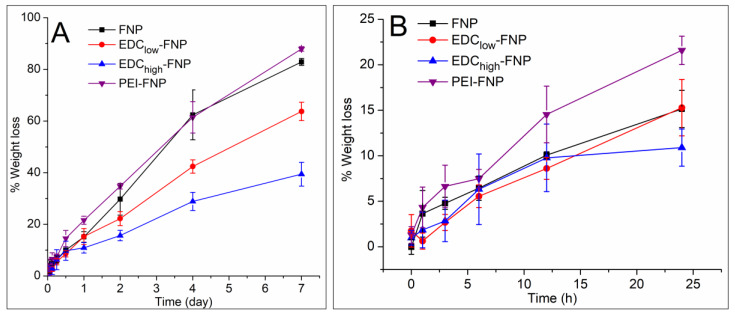
Biostability profiles based on the percentage of weight loss of various fibroin nanoparticle formulations (**A**) during 7-day incubation and (**B**) in the first 24-h incubation (n = 3): The particle biostability was proportionally followed the order of crosslink.

Secondly, FNP degradation, in terms of percentage weight loss, was investigated in the whole blood during a period of 7 days (Figure 2). In the first 12 h, the average weight loss of all formulations was 5–7%, with no significant differences between them (Figure 2B). Interestingly, after 12 h and until 7 days, the degradation rate followed FNP ≈ PEI-FNP > EDC_low_-FNP > EDC_high_-FNP. In general, FNP are degraded by proteolytic enzymes (i.e., proteases), in which most of them mainly cleave the amorphous, less crystalline, or non-compact regions of fibroin [39,40]. Therefore, higher crystallinity EDC_high_-FNP (i.e., more compact structure) [24] was more stable than lower ones, such as EDC_low_-FNP, PEI-FNP, and FNP. Notably, since all formulations possessed similar mean particle size and FITC-binding capacities, FNP polymorph was the main factor affecting FNP degradation rate. Our results suggested that the degradation rate of FNP can be controlled favorably to fit in the biomedical applications by adjusting the particle crystallinity through crosslinking reactions. For example, highly crosslinked EDC_high_-FNP with slow degradation rate can be used in clinical settings that required long treatment duration (i.e., cancer) to reduce the drug administration frequency. On the other hand, the fast degradation rate FNP can be utilized in short-duration treatments.

In terms of hemolysis actions, non-crosslinked FNP have been proved to be nontoxic to the blood [41]. On the other hand, the positively charged particles (i.e., EDC_high_-FNP and PEI-FNP) might theoretically be more toxic to the erythrocytes than the negatively charged particles (i.e., non-crosslinked FNP and EDC_low_-FNP) [31,32]. Surprisingly, our results indicated that all formulations showed no potential toxicity to the red blood cells at a concentration of as high as 1 mg/mL. These data demonstrated that the crosslinked FNP are safe and suitable for systemic applications.

### 2.3. In Vitro Cytotoxicity

In this study, three representative cell lines, Caco-2, HepG2, and 9L, were chosen. Caco-2 is an intestinal cell line, which is beneficial in the study context of an oral exposition to the nanoparticles, whereas HepG2 is a hepatic cell line, which is crucial for intravenous exposition. Additionally, 9L, a gliosarcoma cell line, was used for the potential applications of FNP in cancer treatment.

**Figure 3:**
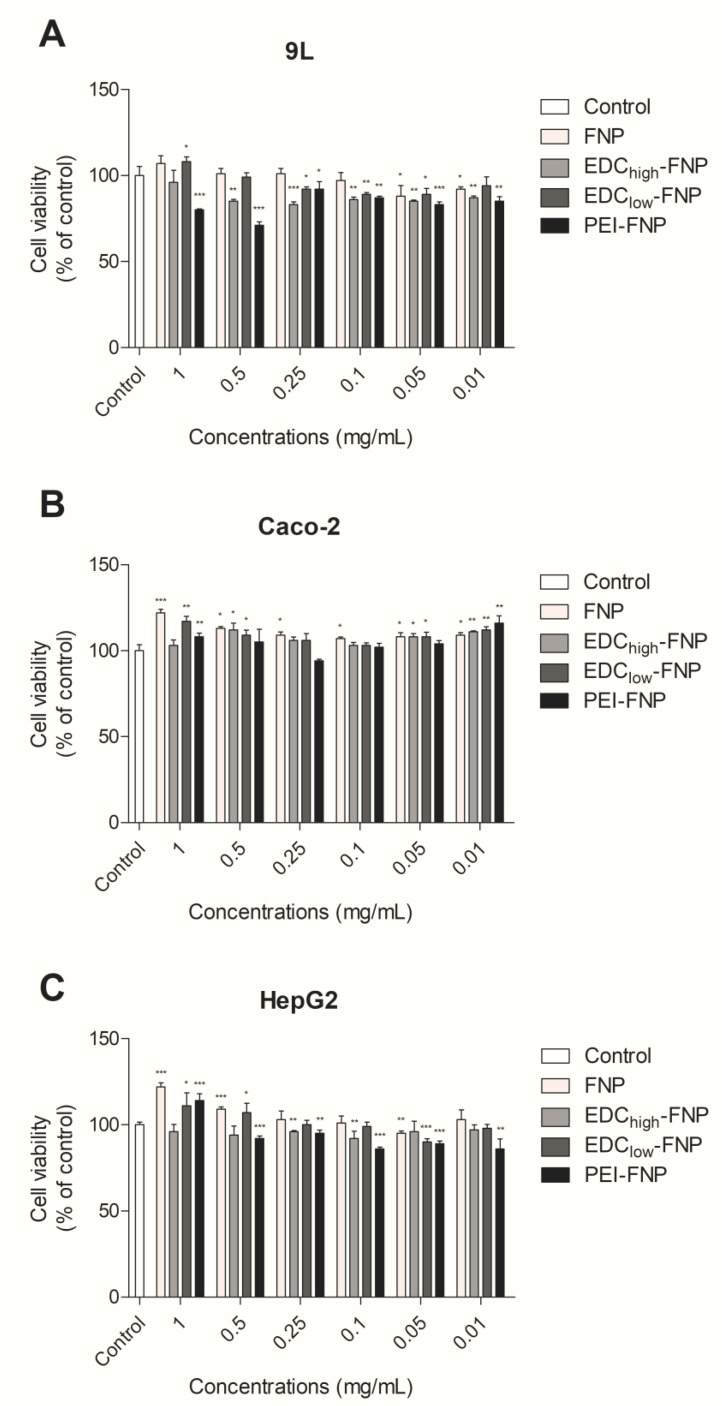
Effect of FNP, EDC_high_-FNP, EDC_low_-FNP, and PEI-FNP (0.01 to 1 mg/mL) on cell viability in 9L (**A**), Caco-2 (**B**), and HepG2 (**C)** cells cultures after 24 h of exposure: Cell viability was determined by an 3-(4,5-dimethylthiazol-2-yl)-5-(3-carboxymethoxyphenyl)-2-(4-sulfophenyl)-2H-tetrazolium (MTS) assay and expressed as a percentage of control. Data are means ± SD from triplicate. * *p* ≤ 0.05, ** *p* ≤ 0.01, and *** *p* ≤ 0.001 with respect to the control.

All cell lines were exposed to FNP at various concentrations (0.01 to 1 mg/mL) for 24 h. The cell viability was then determined by 3-(4,5-dimethylthiazol-2-yl)-5-(3-carboxymethoxyphenyl)-2-(4-sulfophenyl)-2H-tetrazolium (MTS) assay (Figure 3). For the Caco-2 and HepG2 cells, the cell viability was > 80%, regardless of the concentration tested, suggesting the lack of cytotoxic effect of all FNP formulations. However, in 9L cells, only PEI-FNP at high concentrations (1 and 0.5 mg/mL) showed a significant decrease in cell viability, < 80%, while the other FNP formulations showed cell viability of > 80%. This result was in agreement with previous report on PEI-incorporated poly(acrylamide-co-N-(3-aminopropyl) methacrylamide) (PAA) nanoparticles, in which a nanoparticle concentration of > 0.5 mg/mL decreases 9L cell viability [42]. Nevertheless, the free PEI possesses twofold more cytotoxic to 9L cells than the particles [42], indicating the reduction of its toxicity when formulating as PEI-FNP.

### 2.4. Cellular Uptake and Flow Cytometry Study

Cellular interactions between FNP and investigated tissues are crucial for biomedical applications. For example, negatively charged particles might get internalized by the cells less than positively charged ones [32]. Therefore, to investigate the effects of FNP properties on cellular internalization, both qualitatively and quantitatively, the cellular uptake and flow cytometry studies were conducted on the representative Caco-2 cell line.

**Figure 4:**
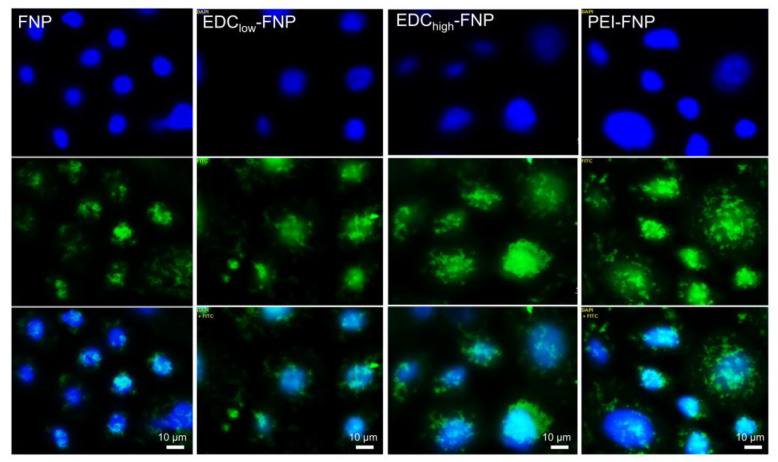
Fluorescence images of Caco-2 cells treated with fibroin nanoparticles after 3-h incubation; showing that all formulations were internalized by the cells: (top) nucleus 4′,6-diamidino-2-phenylindole (DAPI) signal, (middle) particles’ FITC signal, and (bottom) merged images. Scale bar: 10 µm.

**Figure 5:**
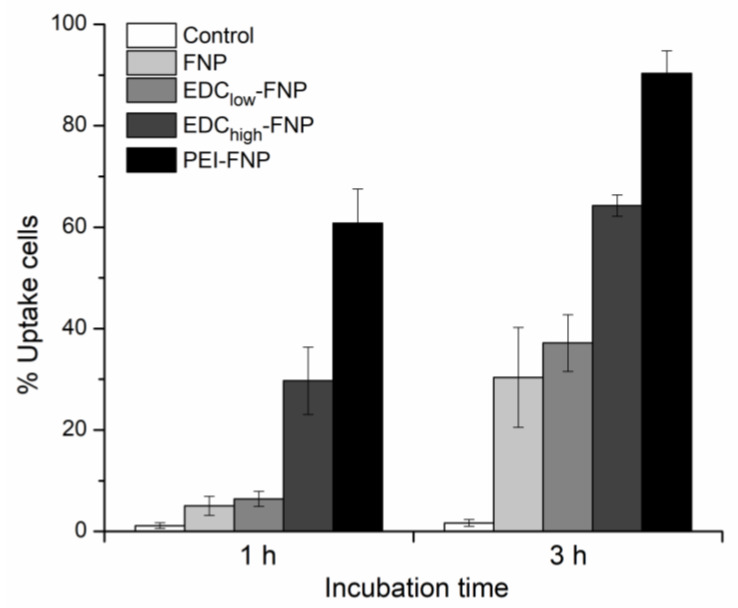
Cellular uptake based on flow cytometry quantitative analysis showing percentage of uptake cell after 1- and 3-h incubation of Caco-2 cells with fibroin nanoparticles; the untreated cell was used as a control. The results are expressed in mean ± SD (error bars) (*n* = 3).

Figure 4 illustrates the fluorescence images of various FITC-bound FNP formulations on Caco-2 cells. Clearly, after 3 h of incubation, regardless of their differences in the zeta potential and crystallinity, all formulations were internalized into the cell cytoplasm and, possibly, nucleus. Furthermore, flow cytometry results clarified that the cellular uptake was time dependent, with significant differences in the percentage of uptake cells on all formulations (Figure 5). Interestingly, the amount of uptake cells varied based on formulations. After 3 h of incubation, the negatively charged non-crosslinked FNP and EDC_low_-FNP showed significantly less uptake compared to the positively charged EDC_high_-FNP and PEI-FNP (approximately 30%, 30%, 60%, and 90%, respectively). This might be explained by the cell–FNP interaction mechanisms. Cellular internalization commonly starts with the nanoparticle adhering on the cell membrane via various forces, such as van der Waals, electrostatic, hydrophobic, and ligand-receptor, before internalization. Thus, stronger forces result in better cell–FNP interactions and, therefore, increased endocytosis. As both EDC_high_-FNP and PEI-FNP possessed positive surface charges, they bound more strongly and efficiently to the negatively charged Caco-2 cell membrane surface, consequently enhancing internalization. Additionally, the PEI branched structure could penetrate and provide more contact points with the cell membrane, further increasing the PEI-FNP uptake. Another issue to note is that the soft (i.e., less tight) particles tend to be internalized more than rigid ones [43]. To this end, PEI-FNP, with lower crystallinity and softer structure [24], was taken up better than the higher crystallinity and rigid EDC_high_-FNP. 

Although being highly internalized by the cells than negatively charged particles, the positively charged particles could possibly never get into contact with cells in tissues that contained a thick mucus layer such as the intestines because they are stuck in this mucosa [30]. Thus, choosing the right FNP properties for suitable administration routes should be greatly considered, as each formulation might interact differently with the biological systems. Our data also suggested that the uptake rate could be altered favorably, dependent on therapeutic applications, based on the particle structure and surface modifications.

## 3. Materials and Methods

### 3.1. Materials

*Bombyx mori* silkworm cocoons were collected from Bodin Thai Silk Khorat Co., Ltd., Nakhon Ratchasima, Thailand. EDC, branched PEI (molecular weight 25,000 Da), FITC, and 4′,6-diamidino-2-phenylindole (DAPI) were bought from Sigma-Aldrich, Singapore. Sheep whole blood was supplied by Nanomed Co., Ltd., Bangkok, Thailand, and were enclosed in a blood-collecting pack containing anti-coagulant CPDA-1 solution (Citrate-Phosphate-Dextrose-Adenine, USP/NF) imported from Fresenius Kabi AG., Bad Homburg, Germany. Other chemicals used are of analytical grade or higher.

For cell culture experiments, Caco-2, HepG2, and 9L cell lines were obtained from American Type Culture Collection (ATCC), with passages of 47, 7, and 3, respectively. Dulbecco’s Modified Eagle Medium (DMEM) Gluta-Max, fetal bovine serum (FBS), phosphate buffered saline (PBS), streptomycin/penicillin (PenStrep), and 0.25% trypsin-ethylenediaminetetraacetic acid (trypsin-EDTA) were purchased from Gibco, Life Technologies Corporation, Waltham, MA, USA. Nonessential amino acid solution (NEAA, 100x) was bought from Sigma-Aldrich.

### 3.2. Fibroin Extraction

Fibroin was extracted from the silkworm cocoons as previously described [24,29]. The cocoons were degummed with 0.5% (*w*/*v*) Na_2_CO_3_ at 100 °C for 1 h, washed thrice with de-ionized (DI) water, air dried, and dissolved again in heated (85–90 °C) mixture of CaCl_2_:H_2_O:Ca(NO_3_)_2_:EtOH (30%:45%:5%:20% *w*/*w*/*w*/*w*). The solution was then dialysed against DI water and lyophilized (Heto PowerDry LL3000, Thermo Fisher, Waltham, MA, USA) at −55 °C, and the obtained fibroin powder was stored at −20 °C.

### 3.3. FNP Formulation

Both non-crosslinked and crosslinked FNP were prepared using desolvation method [24]. Fibroin aqueous solution (1 mL, 1% *w*/*v*) was injected into 0.5 mL EtOH without EDC or PEI (FNP) and with 1% PEI *w*/*v*, pH 7.0 (PEI-FNP), or with EDC (fibroin:EDC ratio of 1:200 mol/mol for EDC_low_-FNP, and 1:1000 mol/mol for EDC_high_-FNP). The spontaneously formed nanoparticles were then stabilized at 4 °C for 24 h, washed thrice with DI water by centrifugation at 31,514 × g (Mikro 220R, Hettich, Germany) for 30 min, and re-dispersed in DI water by sonication (40% amplitude, 30 s). The final products were frozen and lyophilized at 1 × 10^−4^ Torr and −55 °C. The obtained lyophilized powders were stored at 2–8 °C for further experiments.

To visualize the FNP in cellular uptake and flow cytometry studies, the fluorescent dye FITC was used to label the FNP to yield FITC-bound FNP (FITC-FNP). To this end, 100 µL of FITC solution (1 mg/mL in dimethyl sulfoxide (DMSO)) was added dropwise into the FNP dispersions in carbonate buffer, pH 9.0 (20 mg FNP/mL), and stirred at 4 °C for 24 h. To remove the unbound FITC, the mixtures were centrifuged at 31,514 × g for 30 min and re-dispersed in DI water. The process was repeated until no FITC was determined in the supernatant. All formulations were prepared freshly before experiments.

### 3.4. FNP Characterization

#### 3.4.1. Particle Size and Zeta Potential

Dynamic light scattering (DLS) and phase analysis light scattering (PALS) methods (ZetaPALS^®^ analyzer, Brookhaven Instrument Corporation, Holtsville, NY, USA) were used to determine the mean particle size, size distribution (PI), and zeta potential, respectively. For DLS, the instrument was run at 632.8 nm with a BI-200SM Goniometer (Brookhaven Instrument Corporation, Holtsville, NY, USA) connected to a BI-9010AT digital correlator at a fixed angle of 90°. For PALS, each run consisted of 10 cycles at an angle of 14.8° to the incident light. The zeta potential was calculated from the electrophoresis mobility based on the Smoluchowski equation included in the system software. Developed by Marian Smoluchowski in 1903, this equation is the most commonly used theory for calculating zeta potential from experimental data as it is suitable for nanoparticles of any shape and concentration [44]. The FITC-FNP were also imaged using a fluorescence microscope (Axio Observer Z1 model, Carl Zeiss, Oberkochen, Germany). All measurements were determined in triplicate.

#### 3.4.2. FITC-Binding Efficiency

An indirect method was used to determine the amount of FITC that bound to FNP. The supernatants after each washing step were collected and combined, followed by measuring the fluorescence intensity by a fluorescence microplate reader (Synergy H1 Hybrid Reader, BioTek, Winooski, VT, USA) at excitation (Ex) and emission (Em) wavelengths of 490 nm and 525 nm, respectively. The unbound FITC concentrations were calculated using a calibration curve (range: 0.25–20.00 ng/mL, y = 0.0004x + 0.2444, x: FITC concentrations, y: fluorescence intensity, R^2^ = 0.9983). The binding efficacy was determined using Equation (1).

(1) $$\mathrm{Binding}\;\mathrm{efficacy}\;\left(\%\frac{w}{w}\right)=\frac{\mathrm{FITC}\;\mathrm{initial}\;\mathrm{amount}\;\left(100\;µg\right)-\mathrm{Unbound}\;\mathrm{FITC}\;\left(µg\right)}{\mathrm{Amount}\;\mathrm{of}\;\mathrm{FNP}\;\left(20,000\;µg\right)}\;\times \;100\%$$

#### 3.4.3. FITC Dissolution Profile

In vitro dissolution profiles of FITC-FNP were performed using a shaker method. FITC-FNP, equivalent to 30 µg FITC, were dispersed in 50 mL of DI water at 37 °C and stirred at 200 rpm for 8 h. At each time point of 0.5, 1, 2, 4, and 8 h, 1 mL of sample was withdrawn and DI water was refilled. Then, the samples were centrifuged at 31,514 × g for 5 min, and the supernatant containing released FITC was fluorescently measured at Ex/Em = 490/525 nm. The FITC concentrations were calculated using the same calibration curve as mentioned in Section 3.4.2. The cumulative percentage of FITC released at time t (C_t_) was calculated followed Equation (2).

(2) $$\%\;\mathrm{Cumulative}\;\mathrm{release}=\frac{C_{t}V_{0}+V\sum_{1}^{t-1}C_{i}}{M_{0}-\sum_{1}^{t-1}M_{i}}\;\times \;100\%\;$$

where C_t_ and C_i_ are the concentrations of released FITC at the time points t and i, V_0_ is the total volume of dissolution buffer (50 mL), V is the withdrawal sample volume at each time point (1 mL), M_0_ is the initial amount of FITC (30 µg), and M_i_ is the withdrawal total amount of FITC at the time point i.

#### 3.4.4. Physical Stability

To determine the FNP long-term physical stability, the lyophilized powders were stored in a tight container in the dark for 6 months at 4 °C. After 6-month storage, the particle size and zeta potential were determined using ZetaPALS^®^ analyzer.

### 3.5. Biostability Study

To investigate the biostability of FNP in the biological blood, the weight loss experiment was conducted. To this end, 10 mg of each freeze-dried FNP was dispersed in 1 mL of sheep whole blood, followed by continuously shaking at 200 rpm, 37 °C, for 7 days. At each time point of 0, 1, 3, 6, 12, and 24 h and 2, 4, and 7 days, the blood was lysed with 1 mL of DI water and centrifuged at 31,514× *g* for 5 min. The precipitated particles were collected, re-dispersed in DI water, and centrifuged (washing steps) repeatedly until the supernatant was clear. The precipitates were then dried at 60 °C until constant weight. The remaining FNP weights were determined by an analytical balance, and the percentage of weight loss was calculated based on Equation (3). The respective blood samples with no FNP were used as controls.

(3) $$\%\;\mathrm{Weight}\;\mathrm{loss}\;=\frac{\mathrm{Initial}\;\mathrm{amount}\;\left(10\;\mathrm{mg}\right)-\left(\mathrm{Remaining}\;\mathrm{amount}-\mathrm{Control}\right)}{\mathrm{Initial}\;\mathrm{amount}\;\left(10\;\mathrm{mg}\right)}\;\times \;100\%\;$$

### 3.6. Hemolysis

To investigate the in vitro hemolysis action of FNP, sheep red blood cells were used. Briefly, erythrocytes were collected by centrifugation the sheep whole blood at 2432 × g for 5 min. Then, the cells were washed twice in PBS and reconstituted at a concentration of 1% *w*/*v* (1% hematocrit) in PBS. Consequently, all formulations, at various concentrations, were incubated with 1 mL of the prepared erythrocytes at 37 °C for 30 min, followed by halting the reaction with ice for 5 min. Finally, the mixtures were centrifuged at 2432 × g for 5 min, and the hemoglobin presented in the supernatants was UV-Vis spectroscopically measured at 540 nm. The percentage of hemolysis were calculated following Equation (4).

(4) $$\%\;\mathrm{Hemolysis}=\frac{\mathrm{At}-\mathrm{An}}{\mathrm{Ap}-\mathrm{An}}\;\times \;100\%.$$

where At, An, and Ap are the absorbance values of the test samples, the negative control (PBS), and the positive control (DI water), respectively.

### 3.7. Cell Culture

HepG2 and Caco-2 cell lines were cultivated in DMEM Gluta-Max containing 15% FBS, 1% NEAA, and 100 µg/mL streptomycin/100 UI/mL penicillin (1% PenStrep). For 9L cells, the medium was DMEM containing 10% FBS and 1% PenStrep. All cells were cultured at 37 °C in a 5% CO_2_ humidified atmosphere. The medium was changed every other day. The confluent cells were sub-cultured weekly using trypsin-EDTA 0.25%.

### 3.8. In Vitro Cytotoxicity

The evaluation of the cell viability was assessed using the mitochondrial-based CellTiter 96^®^ AQueous Non-Radioactive Cell Proliferation Assay of Promega (MTS assay) (Charbonnieres, France), following the manufacture’s protocol. Cells were seeded 72 h before the treatments in a 96-well plate at 70,000, 80,000, and 20,000 cells/well for Caco-2, HepG2, and 9L cells, respectively. Subsequently, cells were incubated with FNP at various concentrations from 0.01 to 1 mg/mL for 24 h. Cells were then washed once with phosphate buffered saline and then incubated with fresh culture medium containing tetrazolium compound (MTS) and phenazine methosulfate (PMS) for 2 h at 37 °C. The absorbance was measured at 490 nm with a microplate reader (Synergy HT BioTek, Winooski, VT, USA). Mixture of the medium and MTS/PMS without cells served as a blank, and the untreated cells were the control and represented 100% viability. The percentage of cell viability was calculated based on Equation (5).

(5) $$\%\;Viability=\frac{Absorbance_{sample}-Absorbance_{blank}}{Absorbance_{control}-Absorbance_{blank}}\;\times \;100\%$$

### 3.9. Cellular Uptake and Flow Cytometry Study

Prior to experiments, Caco-2 cells were cultured in a 6-well plate (with coverslips for cellular uptake study), with an initial amount of 150,000 cells/well. The medium was changed every even day until the cells reached 70–80% confluence. Then, cells were treated with FITC-FNP (1 µg/mL, equivalent to FITC) for 3 h for cellular uptake study and for 1 and 3 h for flow cytometry. Subsequently, cells were washed thrice with PBS and 5 mM EDTA pH 5.0 to remove the membrane-bound particles.

For cellular uptake study, cells were then fixed with 2.5% glutaraldehyde in PBS, permeabilized using 0.5% Triton-X 100 in DI water for 15 min, and nuclei counterstained with 300 nM DAPI in PBS for 3 min. Finally, cells were washed twice and the coverslip was mounted onto glass slides using glycerol 70% in PBS as a mounting medium. Untreated cells were used as a control. The slides were observed under fluorescence microscope (Axio Observer Z1 model, Carl Zeiss, Oberkochen, Germany) using 405-nm laser line with a band pass (BP) at 350/470 nm for the blue channel to detect DAPI and a 488-nm laser excitation using a BP 490/525 nm for the green channel to detect FITC.

For flow cytometry study, cells were then harvested by trypsinization and resuspended in PBS for measurements. The percentage of cells internalizing FITC-FNP were determined using a flow cytometer (Guava easyCyteTM5, Merck Millipore, Massachusetts, USA), on an average of 10,000 cells/cycle. The results were analyzed using In-Cytes software installed in the machine (Guava soft 3.2).

### 3.10. Statistical Analysis

All experiments were performed at least in triplicate. For quantitative results, the mean ± SD (standard deviation) was reported. One-way analysis of variance (ANOVA) and Student’s t-test were used for statistical purposes, with the *p*-value of at least < 0.05 for significant comparisons.

## 4. Conclusions

This study investigated the in vitro biological properties of the crosslinked FNP utilizing EDC and PEI, with non-crosslinked FNP as a reference. All formulations showed a mean particle size of 300 nm, an adjustable zeta potential from –20 to + 30 mV, and a long-term stability of > 6 months. Additionally, all FNP were biostable in biological systems and showed no potential cytotoxicity to the red blood cells, 9L, Caco-2, and HepG2 cells. Depending on their crystallinity and/or surface modification, FNP degradation rates and cellular internalization rates were controlled favorably. Taking into account of their safety and tunable properties, these crosslinked FNP can be useful in various biomedical applications for both parenteral and non-parenteral routes.

## Figures and Tables

**Table 1:** Mean particle size, polydispersity index, and zeta potential of blank fibroin nanoparticles (FNP) and FITC-bound FNP: The results are expressed in terms of mean ± SD, *n* = 3.

Formulation	Particle Size (nm)	Polydispersity Index	Zeta Potential (mV)
Blank particles			
FNP	282.1 ± 15.0	0.13 ± 0.02	−17.54 ± 0.63
EDC_low_-FNP	289.4 ± 11.1	0.16 ± 0.01	−18.10 ± 0.93
EDC_high_-FNP	310.1 ± 10.5	0.14 ± 0.01	−26.79 ± 0.88
PEI-FNP	305.2 ± 12.8	0.16 ± 0.02	−29.32 ± 1.05
FITC-bound particles			
FNP	300.7 ± 12.6	0.12 ± 0.01	−17.32 ± 0.96
EDC_low_-FNP	296.4 ± 15.3	0.16 ± 0.02	−18.21 ± 1.31
EDC_high_-FNP	292.3 ± 13.4	0.14 ± 0.02	+27.21 ± 1.07
PEI-FNP	311.0 ± 10.9	0.15 ± 0.03	+30.59 ± 1.23

Note: EDC: 1-ethyl-3-(3-dimethylaminopropyl) carbodiimide hydrochloride; PEI: poly (ethylenimine).
